# Supplementary material for: Extreme heterogeneity of influenza virus infection in single cells
Source: eLife. 2018 Feb 16;7:e32303. doi: 10.7554/eLife.32303 (PMC5826275; doi:10.7554/eLife.32303)
Supplement: Figure 5—source data 2. [file elife-32303-fig5-data2.zip › elife-32303-fig5-data2-v2.html]

simple\_Poisson\_model


# Table of Contents

- Heterogeneity in viral infection expected under simple Poisson model
  - Overview of model assumptions
  - Detailed model
  - Defining the model parameters
  - The expected distributions for each sample under the simple model
  - Conclusions

# Heterogeneity in viral infection expected under simple Poisson model¶

This Jupyter notebook contains an analysis of the heterogeneity expected under some simple assumptions for the paper "Extreme heterogeneity of influenza virus infection in single cells" by Alistair Russell, Cole Trapnell, and Jesse Bloom.

## Overview of model assumptions¶

We build a simple Poisson model for the expected heterogeneity under the following assumptions:

1. Infection is a Poisson process
2. Cells infected with a single virion with the full RNP express a given number of viral mRNAs, and cells infected with a single virion lacking an RNP gene express another number of viral mRNAs.
3. Cells that are co-infected simply express a number of viral mRNAs proportional to the number of infecting virions. For instance, doubly infected cells express twice as much viral mRNA as singly infected cells.
4. Each cell also has a fixed amount of cellular mRNA, and the amount of viral mRNA is added to this.

Note that this is a simple model that **we do not think accurately reflects reality.**
The goal of this model is to determine how much heterogeneity we expect under the simplest scenario.

## Detailed model¶

Let a fraction $x\_{full}$ of the virions have the full vRNP, implying that fraction $1 - x\_{full}$ lack an RNP gene.

Let the $\langle v \rangle$ be the mean number of virions of any type (full or partial RNP) that infects the typical cell.
Since we assume infection is Poisson, the distribution of the number of full-RNP virions $v\_{full}$ per cell is $$\Pr\left(v\_{full}\right) = e^{\langle v \rangle x\_{full}} \times \frac{\left(\langle v \rangle x\_{full}\right)^{v\_{full}}}{v\_{full}!}$$
and the distribution of the number of partial-RNP virions $v\_{partial}$ per cell is
$$\Pr\left(v\_{partial}\right) = e^{\langle v \rangle \left(1 - x\_{full}\right)} \times \frac{\left(\langle v \rangle \left(1 - x\_{full}\right)\right)^{v\_{partial}}}{v\_{partial}!}.$$
Since infection is a Poisson process, the probability that a cell is infected by $v\_{full}$ full-RNP virions and $v\_{partial}$ partial-RNP virions is simply
$$\Pr\left(v\_{full}, v\_{partial}\right) = \Pr\left(v\_{full}\right) \times \Pr\left(v\_{partial}\right).$$

Let each cell have $n\_{cell}$ cellular mRNAs.
Let each full-RNP virion add $n\_{full}$ viral mRNAs, and let each partial-RNP virion add $n\_{partial}$ viral mRNAs.
The overall fraction $f\_{viral}$ of mRNA from virus cell if a cell is infected by $v\_{full}$ full-RNP virions and $v\_{partial}$ partial-RNP virions is simply
$$f\_{viral} = \frac{v\_{full}n\_{full} + v\_{partial}n\_{partial}}{n\_{cell} + v\_{full}n\_{full} + v\_{partial}n\_{partial}}.$$

We then simply want to determine the distribution of $f\_{viral}$ across cells among all cells that are infected (have $v\_{full} + v\_{partial} > 0$.

Below we define the functions described above:

In [1]:

```
import math

def f_viral(n_cell, n_full, n_partial, v_full, v_partial):
    """Total fraction of mRNA from virus"""
    numerator = (v_full * n_full + v_partial * n_partial)
    return numerator / float(numerator + n_cell)

def Pr_virions(v_mean, x_full, v_full, v_partial):
    """Probability `v_full` and `v_partial` full and partial virions infect cell"""
    full_mean = v_mean * x_full
    pr_full = math.exp(full_mean) * (full_mean)**v_full / math.factorial(v_full)
    part_mean = v_mean * (1 - x_full)
    pr_part = math.exp(part_mean) * (part_mean)**v_partial / math.factorial(v_partial)
    return pr_full * pr_part
```

## Defining the model parameters¶

We define the model parameters based on the data in our experiments.
Most of these parameters we define separately for each of the three samples (*10hr*, *8hr*, and *8hr-2*) that have substantial amounts of infection.
The actual parameters are in the cell below.

First, we estimate $\langle v \rangle$.
In our experiments, among the cells that are infected, we observe about 10\% of them to have mixed viral barcodes.
Our mixed barcodes will detect about half the co-infections, so we estimate that infected cells have about a 20\% chance of being co-infected.
Based on this, we estimate $\langle v \rangle = 0.2$, which means that the typical cell gets about 0.2 virions (this is essentially the estimate by assuming that all cells are either singly or doubly infected, and neglecting higher-order infections, which is reasonable since most cells are not multiply infected).
Note that this estimate is not entirely consistent with the fraction of cells that exhibit any evidence of infection, indicating the assumption of Poisson infection is unlikely to be fully true.
But we are building the model under that assumption.

We define $x\_{full}$ differently for each sample based on the observed fraction of infected cells with full RNP complements.

To set $n\_{cell}$, $n\_{full}$, and $n\_{partial}$, note that ultimately the units on these does not matter as they are simply used to calculate the fraction $f\_{viral}$.
So we simply set $n\_{cell} = 1$.
We then estimate $n\_{full}$ and $n\_{partial}$ as the median fraction of mRNA from virus among cells infected with the full and partial complement of viral genes.
The rationale here is that most cells are infected with just a single virion, and so the median cell with a full RNP complement got just one full-RNP virion, and the median cell with a partial RNP complement got just one partial-RNP virion.
This justifies setting these numbers as the median fractions of mRNA from virus (assuming $n\_{cell} \gg n\_{full}, n\_{partial}$ so that we ignore the inflation in total mRNA due to viral infection); it would not be justified to set them as the means as the mean would be influenced by co-infections.
The estimates differ across timepoints (as expected since more viral mRNA will be made at later times).

In [2]:

```
samples = ['10hr', '8hr', '8hr-2']

params = {
    'v_mean':0.2,
    'n_cell':1.0,
    '10hr':{
        'x_full':0.67,
        'n_full':0.11,
        'n_partial':0.0051,
        },
     '8hr':{
        'x_full':0.43,
        'n_full':0.016,
        'n_partial':0.0014,
        },
     '8hr-2':{
        'x_full':0.42,
        'n_full':0.042,
        'n_partial':0.0011,
        }
    }
```

## The expected distributions for each sample under the simple model¶

Now we plot the expected distribution of viral mRNA per cell under the simple model.
Rather than trying to solve the model exactly, we just enumerate over all combinations of possible infections allowing up to 20 virions per cell (which is far more than expected to occur at any non-infinitesemial frequency with a Poisson infection process and a mean of $<$1).

We calculate the Gini coefficient using the IneqPy package.

In [3]:

```
import itertools
import numpy
from ineqpy import gini
%matplotlib inline
import seaborn
import matplotlib.pyplot as plt

vmax = 20

for s in samples:
    print("\nPerforming analysis for sample {0}".format(s))
    weights = []
    viralfracs = []
    for (v_full, v_partial) in itertools.permutations(range(vmax + 1), 2):
        if v_full == v_partial == 0:
            continue
        weights.append(Pr_virions(params['v_mean'],
                                  params[s]['x_full'], 
                                  v_full, 
                                  v_partial))
        viralfracs.append(f_viral(params['n_cell'],
                                  params[s]['n_full'],
                                  params[s]['n_partial'],
                                  v_full,
                                  v_partial))
    weights = numpy.array(weights)
    viralfracs = numpy.array(viralfracs)
    
    ginicoeff = gini(income=viralfracs, weights=weights)
    print("The Gini coefficient is {0:.2f}".format(ginicoeff))
    
    print("Here is a plot of the distribution:")
    seaborn.distplot(viralfracs, kde=False, bins=50, hist_kws={'weights':weights})
    plt.xlabel('fraction of mRNA from virus among infected cells')
    plt.show()
```

```
Performing analysis for sample 10hr
The Gini coefficient is 0.77
Here is a plot of the distribution:
```

```
Performing analysis for sample 8hr
The Gini coefficient is 0.75
Here is a plot of the distribution:
```

```
Performing analysis for sample 8hr-2
The Gini coefficient is 0.87
Here is a plot of the distribution:
```

## Conclusions¶

The simple model can generate highly heterogeneous distributions, with Gini coefficients as large or larger than those observed in the real data.
However, the simple model still does not capture the continuous skew observed in the real data.
Under the simple model there are two main peaks: cells infected with a virion lacking RNP, and cells infected with a virion with full RNP.
There are then a few more minor peaks for double infections, and almost nothing beyond that since the MOI is so low.

In contrast, in the real data there is very broad spread, indicating additional processes layered on top of these sources of variability.
Specifically, there must be additional viral, host, or stochastic factors that also spread out the amount of viral mRNA produced even in cells that do or do not receive the full RNP complement of genes.

In [ ]:

```

```
